# PROMIS-9 UE physical function demonstrates moderate responsiveness for patients following upper limb prosthesis intervention

**DOI:** 10.1186/s41687-025-00843-y

**Published:** 2025-02-10

**Authors:** Todd J. Castleberry, Dwiesha L. England, Bretta L. Fylstra, Phillip M. Stevens, Amy E. Todd, Stephen A. Mandacina, Shane R. Wurdeman

**Affiliations:** 1 Hanger Institute for Clinical Research and Education, 10910 Domain Dr., Ste. 300, Austin, TX 78758 USA; 2 Division of Physical Medicine and Rehabilitation, University of Utah Health, Salt Lake City, UT USA; 3 Upper Limb Program, Hanger Clinic, San Antonio, TX USA

**Keywords:** Upper-limb prosthesis, Patient-reported outcomes, Physical function, Prosthesis receipt

## Abstract

**Background:**

Upper extremity physical function is an essential health domain in the rehabilitation care for patients with upper limb amputation or absence. The PROMIS-9 UE Physical Function short form is a recently established instrument designed for individuals with upper limb amputation or absence. The instrument’s responsiveness to changes after receiving a prosthesis has not been investigated. The current study aimed to evaluate the ability of the PROMIS-9 UE to detect changes in bimanual (two-handed) functional capacity after patients’ receipt of a prosthesis.

**Methodology:**

A retrospective chart review was conducted on the longitudinal PROMIS-9 UE outcome measure scores completed between April 2016 and February 2024. Participants included individuals with an outcome collected before and after prosthesis receipt.

**Results:**

The final sample size included 124 individuals (91 male, 33 female; 43.4 ± 15.0 years old, 34.4 ± 103.0 months since amputation, and 62.9% injury etiology). Analyses found significant improvement across all patients in the PROMIS-9 UE scores from baseline to post prosthesis intervention (baseline: 25.3 ± 8.6, post: 29.5 ± 9.6; *p* < 0.001). The PROMIS-9 UE demonstrated a moderate responsiveness (Standardized Response Mean = 0.6) to prosthetic intervention. This was a directional change consistent with subjective patient reports of increased functionality. Furthermore, findings from the linear mixed model demonstrated significant responsiveness for the PROMIS-9 UE instrument to detect post-intervention changes after controlling for potential confounding effects (*p* < 0.001).

**Conclusions:**

These findings suggest the PROMIS-9 UE Physical Function instrument demonstrates moderate clinical utility in capturing patient progress following upper limb prosthesis intervention.

## Background

Amputation of the upper limb can greatly diminish overall functionality [4, 25]. Restoring physical capability is a crucial component of prosthetic rehabilitation for those who have undergone an upper limb amputation [20]. A well-executed prosthetic rehabilitation plan has the potential to restore such functions, especially with the improvements in prosthetic technology [24]. However, insufficient prosthesis use may lead to declines in upper limb strength, endurance, mobility, and could lead to an increase in phantom limb pain [20]. Consequently, for prosthetic rehabilitation to be effective, it necessitates continuous monitoring and adjustments to the care regimen, allowing individuals to realize their full potential [17].

Effective implementation of patient reported outcomes in patient care benefits from psychometrically sound instruments to help capture the patient’s health status [1, 8]. One methodology that was created to produce high-quality instruments to quantify various domains of health and wellness, including physical function, was the Patient- Reported Outcomes Measurement Information System (PROMIS^®^) [2, 22]. Previous work with the PROMIS Upper Extremity Computer Adaptive Test (PROMIS-UE CAT) demonstrated high responsiveness to change in a population of patients with hand or upper extremity injury [9]. However, the limitations of the above findings are that prosthesis users were not noted as part of the target population, and therefore improvement based on the provision of a prosthesis was not investigated. Although the PROMIS-UE has been used to effectively evaluate patients with trauma over time [12, 18], its ability to track responsiveness to prosthetic intervention is unclear.

The PROMIS-UE was created to measure general upper extremity physical function [7]. As a result, its use with prosthetic interventions may not be optimized. The principles of item response theory afforded the ability to customize the specific items from the PROMIS-UE item bank to target individuals with upper extremity amputation/limb difference [11]. Previously, we developed a custom short-form to measure bimanual physical function in prosthesis users (PROMIS-9 UE) [4]. The instrument was tested and demonstrated adequate psychometric properties including validity, reliability, and differential item functioning [4] This custom short form was also found to be a strong predictor of general well-being among prosthesis users [23]. However, there is a lack of understanding on the responsiveness of the PROMIS-9 UE to detect changes following implementation of a new prosthetic intervention among patients with upper limb amputation or absence.

The current study aimed to evaluate the ability of the PROMIS-9 UE to detect changes in bimanual (two-handed) functional capacity after patients’ receipt of a prosthesis. Based on previous findings showing high degree of responsiveness of a PROMIS physical function instrument among individuals without upper limb amputation, we hypothesized that the PROMIS-9 UE would also demonstrate a moderate to high level of responsiveness among patients using upper limb prostheses.

## Methods

### Participant and study design

A retrospective chart review was conducted on a longitudinal clinical outcomes database completed between April 2016 and February 2024. The database included upper limb prosthesis users presenting at a system of private prosthetics clinics across the United States. As part of routine clinical care, clinicians collected demographic information and patients reported outcomes measures, including the PROMIS-9 UE. The PROMIS-9 UE was completed before prosthesis receipt, and again between two weeks and twelve months after the receipt of their prosthesis. Respondents were included in the final analysis if they were 18 years and older. The minimum timeframe from the provision of their prosthesis to follow-up was set to two weeks, similar to previous studies [3, 6]. There were no exclusions of patients based on amputation level, etiology, or prosthesis type. The Western Copernicus Group Institutional Review Board (protocol number 20170059) approved and deemed this study exempt from informed consent.

### Instrument

The primary outcome instrument utilized in this analysis was the PROMIS-9 UE physical function custom short form [4]. The PROMIS-9 UE instrument consists of nine items (PFA20, PFA28, PFA35, PFA54, PFB20r1, PF27, PF30, PFM2, and PFM16) selected by content experts from the PROMIS v2.0 Upper Extremity Item bank. These items were administered in English using paper surveys to evaluate the patients’ perceived ability to complete bimanual tasks such as using eating utensils, opening a can with a hand can opener, cutting paper with scissors, etc. The response categories range from “unable to do” to “without any difficulty”, with a corresponding response score from one to five. Item PB20r1 is an exception with PROMIS developers collapsing the score range from one to four [2]. A recent study reported acceptable psychometric properties of the PROMIS-9 UE in a population of individuals with upper limb amputation [4].

The PROMIS-9 UE raw scores were converted to T-scores using the HealthMeasures.net scoring service. The PROMIS-9 UE T-score was used as the dependent variable in the models. It is worth noting PROMIS developers calibrated the PROMIS Upper Extremity Item bank using a sample of individuals with and without an upper extremity injury, with higher scores indicating greater physical function levels. The possible range of scores are between 9.1 and 60.3. Subsequently, it should be expected that individuals with increased functional impairment, such as with upper limb amputation, will report on average lower than the population mean of 50.0.

The main independent variable was relative to time of prosthesis intervention: before and after prosthesis receipt. Other variables included demographic factors such as age, hours worn, gender, amputation group, time since amputation, time between delivery of first prosthesis and follow-up, cause of amputation, and device type. Amputation group was divided into six distinct models: (1) All (all amputation groups), (2) TR/TH/WD/ED (transradial/transhumeral/wrist disarticulation/elbow disarticulation), (3) TH/ED, (4) TR/WD, (5) Bilateral, and (6) PH/PF (partial hand/partial finger). Amputation groups include a combination of amputation levels, and multiple arm involvement, including bilateral.

### Analyses

Frequency, average, and standard deviation were computed for demographic variables at the time of baseline (i.e., before prosthetic rehabilitation). Hours worn was evaluated at follow-up (i.e., after prosthetic rehabilitation). Similar to Husted [10], a one-tailed paired t-test, Standardized Response Means (SRM), and random intercept linear mixed models were used to evaluate the responsiveness. Statistical significance was determined using a threshold of (*p* < 0.05). According to the COSMIN initiative, responsiveness is defined as the instrument’s ability to detect changes over time in the construct being measured [14]. The responsiveness of the PROMIS-9 UE in detecting differences in Physical function before and after prosthesis intervention, stratified by amputation groups was examined. The SRMs were calculated based on Cohen and Liang approach [10]. When interpreting SRM, a value of ≥ 0.2 and < 0.5 represents a small effect, a value of ≥ 0.50 and ≤ 0.80 represents a moderate effect, and a value of > 0.80 represents a large effect. Lastly, the linear mix models were computed using the R package LmerTest [13]. The model was adjusted by entering age, gender, hours worn, amputation group, time between prosthesis receipt and follow-up, and time since amputation into the model as fixed effects. A deidentified subject number was entered as a random effect. P-values for the linear mix model were derived using Satterthwaite’s degrees of freedom test. All statistical analyses were conducted using R 4.1.0 software.

## Results

### Sample demographics

The final sample consisted of 124 individuals meeting the inclusion criteria (Table 1). Of the 124 individuals, 73.4% were male, and 62.9% had an amputation due to trauma/injury. Of those that reported a device type, the most common device was a body-powered prosthesis. The average age was 43.4 ± 15.0 years old with a time since amputation of 34.4 ± 103.0 months. All patients included in this sample had their follow-up time point between two weeks and twelve months with the average being 3.4 ± 3.2 months.

**Table 1:** Patient demographics (*n* = 124)

Patient Demographics	Mean ± SD
Age (yrs)	43.4 ± 15.0
Height (m)	1.8 ± 0.2
Weight (kg)	85.9 ± 20.3
Time since amputation (months)	34.4 ± 103.0
Time between delivery and follow-up (months)	3.4 ± 3.2
Hours worn Daily	6.2 ± 4.9

	**n (%)**
Gender	
Female	33 (26.6)
Male	91 (73.4)
Amputation Group	
Elbow Disarticulation/Transhumeral	18 (14.5)
Wrist Disarticulation/Transradial	28 (22.5)
Partial Finger/Partial hand	71 (57.3)
Bilateral	7 (5.6)
Cause of Amputation	
Vascular	5 (4.0)
Injury	78 (62.9)
Infection	12 (9.7)
Congenital	3 (2.4)
Cancer/Tumor	5 (4.0)
Other/Not Reported	21 (16.9)
Device Type	
Body Powered	48 (38.7)
Electronic Arm	14 (11.3)
Not reported	62 (50.0)

### Univariate modeling: paired T-test and standardized response mean

The paired t-test showed significant improvement in the PROMIS-9 UE scores after prosthesis receipt (Before: 25.3 ± 8.6; After: 29.5 ± 9.6; *p* < 0.001) (Table 2; Model 1). The PROMIS-9 UE demonstrated a moderate degree of responsiveness (SRM = 0.60) for the combined cohort of all amputation groups. Additionally, changes in function from pre and post prosthesis receipt were measured within each model (Table 2).

**Table 2:** Results of standardized response mean and paired t-test comparing baseline and follow-up physical function t-score for different amputation groups

	Amputation Group	*N*	Baseline: Mean ± SD	Follow-up: Mean ± SD	*P*	SRM
Model 1	All	124	25.3 ± 8.6	29.5 ± 9.6	< 0.001	0.60
Model 2	WD/TR/ED/TH	46	23.1 ± 7.8	25.5 ± 8.1	0.009	0.31
Model 3	ED/TH	18	23.9 ± 10.4	26.8 ± 10.8	0.072	0.35
Model 4	WD/TR	28	22.6 ± 5.6	24.7 ± 5.9	0.030	0.36
Model 5	Bilateral	7	18.0 ± 5.8	25.1 ± 5.2	0.001	1.67
Model 6	PH/PF	71	27.5 ± 8.7	32.5 ± 9.7	< 0.001	0.72

SD: standard deviation, SRM: Standardized Response Mean, WD: wrist disarticulation, TR: transradial, ED: elbow disarticulation, TH: transhumeral, PH: partial hand, PF: partial finger

### Multivariate modeling: linear mixed model

Findings from the linear mixed model demonstrated responsiveness for the PROMIS-9 UE instrument to detect changes after receiving the prosthesis (Table 3). After controlling for the possible confounding effect of age, gender, hours worn, amputation group, time between delivery and follow-up and time since amputation, patients’ PROMIS-9 UE increased by 4.2 points (*p* < 0.001; Table 3).

**Table 3:** Results from the multivariate linear mixed-effects regression showing the impact of the first prosthesis intervention on physical function, adjusted for covariates and stratified by amputation groups

Variables	Model 1: All Amputation Groups	Model 2: TR/TH/WD/ED	Model 3: TH/ED	Model 4: TR/WD	Model 5: Bilateral	Model 6: PH/PF
	Estimate [95% Cl]	Estimate [95% Cl]	Estimate [95% Cl]	Estimate [95% Cl]	Estimate [95% Cl]	Estimate [95% Cl]
Prosthesis Intervention						
Before	ref	ref	ref	ref	ref	ref
After	4.18 [2.97, 5.38]	2.45 [0.49, 4.42]	2.94 [-0.93, 6.82]	2.14 [-0.03, 4.32]	7.14 [5.10, 9.19]	5.00 [3.38, 6.62]
Age (years)	-0.10 [ -0.19, -0.02]	-0.08 [-0.21, 0.04]	-0.05 [-0.28, 0.17]	-0.11 [-0.22, -0.01]	0.44 [0.12, 0.75]	-0.12 [-0.25, 0.01]
Hours Worn	0.08 [-0.22, 0.38]	-0.05 [-0.40, 0.29]	-0.32 [ -0.95, 0.31]	-0.04 [-0.33, 0.25]	0.55 [-0.22, 1.32]	0.10 [-0.45, 0.65]
Gender						
Female	ref	ref	ref	ref	ref	ref
Male	-0.16 [-3.29, 2.96]	3.97 [-0.24, 8.17]	-0.43 [-9.92, 9.05]	4.22 [0.71, 7.71]	-13.15 [-23.37, -2.94]	-2.73 [-7.54, 2.08]
Time between delivery and follow-up (months)	-0.09 [-0.51, 0.33]	-0.07 [-0.66. 0.52]	0.23 [-0.83, 1.29]	-0.12 [-0.59, 0.36]	3.02 [1.26, 4.79]	0.06 [-0.59, 0.71]
Time since amputation (months)	0.02 [0.01, 0.03]	0.01 [-0.00, 0.02]	0.10 [ 0.05, 0.14]	0.01 [-0.00, 0.02]	0.01 [-0.02, 0.05]	0.03 [0.00, 0.06]
Amputation group						
ED/TH	ref					
WD/TR	-2.38 [-6.96, 2.19]					
PH/PF	4.77 [0.90, 8.66]					
Bilateral	-3.48 [-10.05, 3.10]					

Comparing the results across the six models, all models were significantly different before versus after receipt of a prosthesis except for Model 3 (transhumeral, elbow disarticulation). The model was not significant (*p* = 0.072). The result may be explained by the small sample size and large variability across participants (standard deviation ~ 10 points). Additionally, Model 4 (transradial, wrist disarticulation) had lower average physical function T-scores compared to Model 3. This contrast to clinical experience where distal amputations have higher physical function may further underscore the large variability in participant responses and smaller sample size in Model 3 limiting the statistical strength.

## Discussion

Results from this study further the value and clinical utility of the PROMIS-9 UE short form for patients with upper limb amputation or absence. Specifically, this study demonstrated moderate responsiveness in the instrument comparing individuals before and after initiation of the patients’ prosthetic rehabilitation with prosthetic intervention. These findings further support the use of the PROMIS-9 UE custom short form for measuring patient progress throughout their prosthesis rehabilitation journey.

A recent study found no difference in physical function between prosthesis users and non-users [21]. This may seem somewhat in contrast to the current study results. However, there are a few factors that may explain. First, Resnik et al. performed a cross-sectional study of affected individuals that had established usage or non-usage patterns with respect to their prostheses versus the current longitudinal assessment of changes in function experienced by an individual adapting to a new resource. In addition, that study compared the PROMIS UE 7-item, 6-item, and 13-item short forms for individuals with unilateral upper limb amputation which included a variety of one-handed and two-handed tasks. By contrast, the PROMIS-9 UE short form focuses exclusively on two-handed tasks.

Another study reported that individuals with unilateral upper limb amputation utilized their prosthesis for 24% of one-handed tasks and 38% of two-handed tasks, suggesting a higher demand for prosthesis use in bilateral activities [19]. The current study further highlights this finding of a higher prevalence of two-handed tasks, emphasized by the PROMIS-9 UE’s focus. This emphasis on two-handed tasks, further enhances the PROMIS-9 UE’s sensitivity in detecting clinical changes in physical function for individuals with unilateral upper limb amputation.

The average T-scores both before and after initiation of prosthesis intervention were more than two-standard deviations below the general population mean of 50 for the PROMIS instrument for upper limb function [16]. This underscores the functional deficits associated with major upper limb amputation, both with and without prosthetic rehabilitation. This may also indicate potential value of future work to transform the PROMIS-9 UE T-score to a scale calibrated to a population of individuals with upper limb amputation and difference.

It is worth noting a few limitations of this work. The time interval between delivery and follow-up varied among participants. There was an attempt to control for this factor in our statistical model, but the variations are an inherent limitation to analyses of real-world clinical outcomes data. We also did not control for type of prosthesis receipt (i.e., body powered versus myoelectric). Therefore, responsiveness within group may not be as clear. Some follow-up visits might have coincided with routine check-ups without any problems, while others might have involved clinical issues (e.g., broken componentry or skin/fit problems with the prosthesis socket). The results showed an average increase in function with prosthesis delivery, but it is not possible to confirm that the patients were at their optimal function. Although establishing population or diagnostic-specific thresholds through “bookmarking” was not the goal of this study, we may consider this approach in future research to establish more precise criteria for thresholds on physical function. Additionally, we were unable to identify patient participation in physical or occupational therapy. Future work is needed to examine the impact of physical or occupational therapy.

## Conclusions

In summary, the current analysis provides evidence that further demonstrates the clinical effectiveness of the PROMIS-9 UE for evaluating upper extremity function in patients who initiate upper limb prosthetic rehabilitation [5, 15]. As such, the custom PROMIS-9 UE is considered to be acceptable regarding clinical responsiveness to prosthesis intervention. The use of PROMIS-9 UE can improve the precision of assessments and promote patient-centered care across the upper limb prosthesis user community.

## Data Availability

All data generated or analyzed during this study are included in this published article.

## Abbreviations

PRO: Patient-reported outcome
PROMIS: Patient-Reported Outcome Measurement Information System
UE: Upper Extremity
PF: Physical Function
SRM: Standardized Response Mean
MCID: Minimal Clinically Important Difference
